# Neural Correlates Predicting Lane-Keeping and Hazard Detection: An fMRI Study Featuring a Pedestrian-Rich Simulator Environment

**DOI:** 10.3389/fnhum.2022.754379

**Published:** 2022-02-09

**Authors:** Kentaro Oba, Koji Hamada, Azumi Tanabe-Ishibashi, Fumihiko Murase, Masaaki Hirose, Ryuta Kawashima, Motoaki Sugiura

**Affiliations:** ^1^Institute of Development, Aging and Cancer, Tohoku University, Sendai, Japan; ^2^DENSO CORPORATION, Kariya, Japan; ^3^International Research Institute of Disaster Science, Tohoku University, Sendai, Japan; ^4^Smart-Ageing Research Center, Tohoku University, Sendai, Japan

**Keywords:** frontoparietal control network, superior temporal sulcus, driving simulator, driving safety, fMRI

## Abstract

Distracted attention is considered responsible for most car accidents, and many functional magnetic resonance imaging (fMRI) researchers have addressed its neural correlates using a car-driving simulator. Previous studies, however, have not directly addressed safe driving performance and did not place pedestrians in the simulator environment. In this fMRI study, we simulated a pedestrian-rich environment to explore the neural correlates of three types of safe driving performance: accurate lane-keeping during driving (driving accuracy), the braking response to a preceding car, and the braking response to a crossing pedestrian. Activation of the bilateral frontoparietal control network predicted high driving accuracy. On the other hand, activation of the left posterior and right anterior superior temporal sulci preceding a sudden pedestrian crossing predicted a slow braking response. The results suggest the involvement of different cognitive processes in different components of driving safety: the facilitatory effect of maintained attention on driving accuracy and the distracting effect of social–cognitive processes on the braking response to pedestrians.

## Introduction

Driving is based on the continuous adjustment and reallocation of attention, which can be affected by various sources of distraction (Palmiero et al., 2019). The principal factor in fatal traffic accidents is distracted attention on the part of the driver (Klauer et al., 2014). For example, in Japan, distracted driving and inattentive driving, which are violations of the duty to drive safely and are directly related to inattention while driving, accounted for, respectively, 14.8% and 11.7% of fatal accidents in 2019 (White Paper on Traffic Safety in Japan, 2020). A large study of driving in actual driving situations (Dingus et al., 2006) determined that inattention was involved in 78% of accidents and 65% of near-accidents. Several studies have investigated the relationship between attention and driving abilities in experimental settings. For example, Hoffman and colleagues (Hoffman et al., 2005) found that attention deficits in older drivers predicted their impairment in simulated driving, as reflected in an increase in the number of car crashes. Cuenen and colleagues demonstrated that the attentional abilities of elder drivers can predict their reaction times to road hazards in a driving simulator task (Cuenen et al., 2015). To reduce traffic accidents, it is important to investigate the relationship between attentional states and safe driving performance.

Safe driving is supported by a variety of driving performances such as longitudinal performance (e.g., velocity, response time, headway distance), lateral performance (e.g., steering operation, time to line crossing), parking maneuver, and situation awareness (Greenlee et al., 2018; Akamatsu, 2019). Although research using driving simulators has begun to explore the neural basis of driving and the effect of attention distraction on them, to the best of our knowledge, there have been no studies that have used the within-subject approach to examine the effects of attentional fluctuations on safe driving. The introduction of driving simulator into functional magnetic resonance imaging (fMRI) has received considerable attention (Walter et al., 2001; Calhoun et al., 2002; Uchiyama et al., 2003; Calhoun and Pearlson, 2012; Kan et al., 2013; Choi et al., 2017), and these studies have revealed the involvement of brain areas such as the sensorimotor cortices and cerebellum as well as the visual cortex, prefrontal cortex, and subcortical areas during simulated driving (Navarro et al., 2018). Numerous experiments using a secondary task during simulated driving to distract attention have been conducted in the last 10 years (Graydon et al., 2004; Just et al., 2008; Hsieh et al., 2009; Uchiyama et al., 2012; Schweizer et al., 2013; Chung et al., 2014), although most of these studies have only examined the effect of the distractor on brain activity during car driving condition. These studies have suggested a significant shift in activation from the occipital to the frontoparietal brain regions under a dual-task condition (simulated driving plus a secondary task) as compared to a simulated driving condition alone (Palmiero et al., 2019). However, relationships between the frontoparietal network and measures of safe driving performance, such as accurate lane-keeping during driving (driving accuracy), have not been investigated. The frontoparietal control network, a robust network mainly comprising the lateral prefrontal cortex (including the rostral and dorsolateral prefrontal cortex) and inferior parietal lobule (Power et al., 2011; Niendam et al., 2012; Cole et al., 2013; Gordon et al., 2016; Gratton et al., 2018; Uddin et al., 2019), is involved in executive functions such as vigilance or sustained attention, the initiation of complex goal-directed behaviors, the inhibition of prepotent but incorrect responses, flexibility in shifting easily between goal states, planning the steps necessary to achieve a goal, and working memory or the ability to hold information in mind and manipulate it to guide response selection (Niendam et al., 2012; Uddin et al., 2019). Furthermore, in the presence of a distractor, numerous studies have reported a decrease in driving performance and an increase in activation in areas related to perceptual processing of the distractor, such as the visual and auditory cortex (Just et al., 2008; Hsieh et al., 2009; Schweizer et al., 2013; Palmiero et al., 2019). However, little information is available about the relationship between brain activity and safe driving performance. A few studies have reported the neural correlates of driving performance at a between-subjects level, such as the involvement of the anterior cingulate cortex in car-following performance (Uchiyama et al., 2003), the bilateral lateral occipital complex, and right inferior parietal lobule activity in car-following performance (Uchiyama et al., 2012), and the right superior parietal lobule in visual multitasking performance (Al-Hashimi et al., 2015). Although the results of between-subjects correlation analysis can reveal the neural correlates of individual differences in driving ability and driving strategy, to clarify the cognitive states related to safe driving, which fluctuate within individuals at the brain level, it is important to examine within-subject correlations based on time series of brain activity and performance.

In most of the studies noted above, the driving environment included landscapes and vehicles but not pedestrians (Walter et al., 2001; Graydon et al., 2004; Just et al., 2008; Hsieh et al., 2009; Uchiyama et al., 2012; Kan et al., 2013; Chung et al., 2014; Choi et al., 2017). Each year, more than 270,000 pedestrians lose their lives on the world’s roads, and globally, pedestrians constitute 22% of all road deaths; in some countries, this proportion is as high as two-thirds (World Health Organization (WHO), 2013). Millions more people are injured in traffic-related crashes while walking, some of whom become permanently disabled (World Health Organization (WHO), 2013). Pedestrians are one type of external distractor (Dingus et al., 2006); furthermore, social information such as that represented by pedestrians captures the driver’s attention in a task-irrelevant manner by eliciting spontaneous mentalizing during simulated driving (Spiers and Maguire, 2006). Thus far, a few fMRI studies have placed pedestrians in the driving simulator, but these have not analyzed brain activity in response to the pedestrians (Spiers and Maguire, 2007; Li et al., 2012).

This study aimed to examine, for the first time, the neural states associated with safe driving by investigating within-subject correlations between brain activity and safe driving performance in a pedestrian-rich environment. We created a driving simulator that contained rich social information and examined the brain activity associated with safe driving performance, including driving accuracy as measured by lane-keeping and the braking response to a preceding car and pedestrians. Because many studies have suggested the involvement of the frontoparietal control network during simulated driving with distractors (Graydon et al., 2004; Li et al., 2012; Kan et al., 2013; Chung et al., 2014) and assessed lane-keeping as moderated by attention capacity (Cuenen et al., 2015), we specifically predicted that frontoparietal control network activity would positively correlate with better lane-keeping.

## Materials and Methods

### Participants

Thirty-eight healthy right-handed participants who had driver’s licenses participated in this study. Inclusion criteria were healthy right-handed young person over the age of 20 and having a driver’s license. The handedness was assessed by Edinburgh inventory (Oldfield, 1971). As for the frequency of driving, 19 participants drove less than once a month, 10 drove 1–2 times a month, one drove one-two times a week, and no participant drove more than three times a week. Exclusion criteria were the presence of metal in the body or on the body surface, a history of psychiatric or neurological disorders, claustrophobia, and the possibility of pregnancy. The experiment was stopped for one participant due to visually induced motion sickness. Four participants were removed from the analysis due to low-quality data (more than 20% of the trials with misses for either the preceding car or the crossing pedestrian). The data of three participants were removed from the analysis due to a technical problem during the simulator presentation. Ultimately, the data of 30 participants were used for the analysis [eight females, age range: 20–38 years old, mean (M) age = 21.9 years, standard deviation (SD) = 3.7 years]. All of them were in college or had more than a college degree. All participants provided written informed consent before their participation. This study was approved by the Research Ethics Committee of Tohoku University School of Medicine and was conducted in accordance with the tenets of the Declaration of Helsinki.

### Task

**Figure 1:**
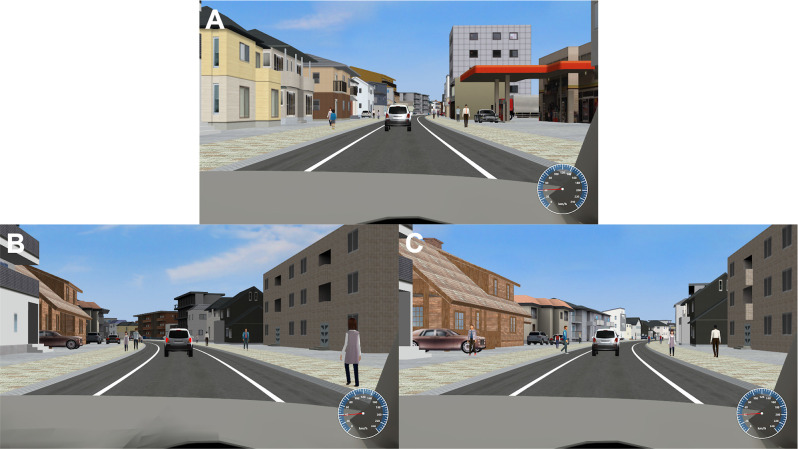
Driving simulator and emergency events. **(A)** While driving along a gently S-shaped road, participants were asked to control the car so that it was positioned in the center of the road as much as possible (driving accuracy) by pushing the left and right buttons of an MRI-compatible response button. They were also asked to perform braking responses to two types of emergency events: **(B)** deceleration of the vehicle ahead (the brake lights of the vehicle ahead would glow) and **(C)** a pedestrian moving from the sidewalk on the right or left toward the road (in this case, a man in a blue shirt is walking toward the road).

We created a task, experienced from a first-person perspective, in which participants continuously drove along a one-way, gently S-shaped road in a city (Figure 1). With pedestrians on the sidewalks on both sides and another car in front of their car, the participants were asked to control their car such that it stayed in the middle of the street as much as possible by pushing left/right buttons using MRI-compatible response buttons (Current Designs; Philadelphia, PA, USA). Each button press made a stepwise change of direction. Two types of situations were set as emergency events while driving, similar to a previous study (Yanko and Spalek, 2013): braking by a preceding car (Figure 1B, the brake lights of the vehicle ahead glow, and the vehicle slows down) and a pedestrian crossing (Figure 1C, heading toward the road from the sidewalk on the right or left). There was always the same one car in front of the driver’s car as shown in Figure 1, and sometimes it braked. The car ahead was set to a distance of 50 m, and the time from deceleration to stop and re-acceleration was set to 4 s. A pedestrian appears 50 m ahead of the vehicle, walks in the crossing direction for 2 s, and stops on the shoulder. Since the speed of the vehicle is 40 km/h, the vehicle reaches the position of the pedestrian in 4.5 s after the pedestrian appears. During an experimental session (16 min, 10 s), preceding car slowdowns or pedestrian-crossing events occurred pseudorandomly 30 times, each at intervals of 15 s or more. Participants were asked to press a button using the right thumb as soon as possible to avoid the dangers (braking response). The reaction time (RT) was calculated as the time from when the brake lights of the preceding car came on to the right thumb button press, and the time from when the pedestrian started moving from the sidewalk to the road to the right thumb button press, respectively. A 500 Hz pure tone was fed back to the participant to indicate the right thumb button press. Successful avoidance was visualized as the own vehicle slowing down or the pedestrian disappearing. The vehicle speed was kept constant at 40 km/h except during temporary deceleration and re-acceleration when the preceding car slowed down. The total number of pedestrians walking on the sidewalk was 840 and that of crossing pedestrians was 30 in each session, therefore the rate of crossing pedestrians was 0.036 (30 divided by 840). This 8indicates that crossing pedestrians were rare and can be dangerous and alarming for the participants.

Six driving simulation scenarios of 3.5 km per cycle (with different timing of occurrence of hazardous events) were created, and three of them were connected in different combinations to create four experimental tasks of 10.5 km each. The time required to drive 10.5 km is about 15 min, but since the time required for the task was slightly extended depending on the participant’s driving performance (steering and braking reaction time), we set the MRI scanning time to 16 min and 10 s per session. The order of task presentation was counterbalanced among the participants. The driving simulator task was created using UC-win/Road Driving Sim Ver.13(SS; FORUM 8 Co., Ltd).

The simulator contained the sound of the car’s engine and a 500 Hz pure tone that indicated the participant’s right thumb button press, and these sounds were presented to the participant using MRI-compatible headphones (Resonance Technology Inc. Northridge, CA, USA). When the car slowed down in response to the braking of the car ahead, the pitch of the engine sound also became lower.

After being briefed on the task, participants performed one session of practice trials (16 min) in the presence of the experimenter; if subjects did not report that they were sufficiently familiar with the task after one session of practice, or if the experimenter could not determine that they were sufficiently familiar with the task, additional practice sessions were conducted.

### fMRI Measurements

Scanning was conducted using a 3 T MRI scanner (Achieva Quasar Dual, Philips). Blood oxygenation level-dependent (BOLD) T2*-weighted MR signals were measured using a gradient echo-planar imaging sequence. Forty 3-mm-thick contiguous slices covering the entire brain were acquired (repetition time [TR] = 2,500 ms, echo time = 30 ms, flip angle = 85°, field of view = 192 mm^2^, and scan matrix = 64 × 64). Excluding the first two “dummy” volumes to stabilize the T1-saturation effect, 388 volumes were acquired in each fMRI session.

### Analysis

The following preprocessing procedures were performed using CONN (Whitfield-Gabrieli and Nieto-Castanon, 2012) implemented in MATLAB R2018a (MathWorks; Natick, MA, USA): realignment and unwarp where the potential susceptibility distortion-by-motion interactions were addressed by estimating the derivatives of the deformation field for head movement and resampling the functional data to match the deformation field of the reference image (first scan of the first session), slice timing correction, outlier detection for scrubbing (intermediate setting: framewise displacement above 0.9 mm or global BOLD signal changes above 5 SD), normalization to Montreal Neurological Institute (MNI) space by unified segmentation and normalization (Ashburner and Friston, 2005) where the mean EPI was segmented, non-linear spatial transformation was conducted, and the resulting deformation field was applied to the EPI time series for normalization, and smoothing using a Gaussian kernel with a full-width at a half-maximum value of 8 mm.

The relationship between brain activity and safe driving performance for each voxel was estimated using a general linear model (GLM). In this study, we employed the time to line crossing (TTLC) and the reaction time (RT) for hazardous events as representatives of driving performance measures (Greenlee et al., 2018; Akamatsu, 2019). To examine the neural correlates of driving accuracy and hazard detection (preceding car and pedestrian), two types of GLM analysis were performed using statistical parametric mapping (SPM12) software (Wellcome Department of Imaging Neuroscience; London, UK). The timing of the outlier scan and realignment parameters were also included in the model to remove the effects of head movement. A high-pass filter (128 s) was used to remove low-frequency noise. Brain areas that exhibited significant relationships with safe driving performance were mapped onto the brain surface and sections using bspmview (Spunt, 2016).

#### (1) Driving Accuracy

As a measure of driving accuracy, the time to line crossing (TTLC) was calculated based on the car position, which was sampled continuously throughout the session. TTLC represents the duration of time available before any lane boundary is crossed; the larger the value, the more accurate the performance (Mammar et al., 2006). Specifically, when the distance to the shoulder on the direction of travel was D [m] and the lateral speed was V [m/s], TTLC was calculated as TTLC = D/V [s]. TTLC time-series data were down-sampled to the minimum value of 2.5 s, which matched the TR value, and then convolved with the canonical hemodynamic response function (HRF) implemented on SPM12 for use as the regressor of interest representing the expected hemodynamic response related to TTLC. In the same way, the road curvature regressor was constructed and incorporated into the model to exclude the possible effect of curvature on brain activity. In addition, the model included the following regressors constructed by convolving delta functions on each event onset and the canonical HRF: pedestrian crossing, preceding car slowdown, button press by the right thumb for hazard detection, button press by the left thumb to turn the wheel to the right, button press by the left thumb to turn the wheel to the left, scenario switch (at around 5 and 10 min), and error (no response to both pedestrian crossing and preceding car slowdown).

To further examine the effects of learning/habituation, the same analysis was performed for the first and second half of each run to examine differences in the frontoparietal control network ROI and whole brain.

#### (2) Braking Response to a Preceding Car and a Crossing Pedestrian

To investigate brain activity that precedes the response to hazardous events, such as a preceding car and a crossing pedestrian, the relationship between the brain activity prior to these events and the reaction time (RT) for each event was investigated using parametric modulation analysis. We chose a time window of 10 s referring to a previous study that examined the neural correlates of mind wandering (Christoff et al., 2009). In that study, the authors used experience sampling to provide an online measure of mind wandering during a concurrent attention task and succeeded in demonstrating that the default network activation in 10 s interval of time immediately preceding each sampling was observed both in association with subjective self-reports of mind wandering and performance errors on the concurrent task. More specifically, in the current study, the time point 10 s before the actual event onset was modeled by convolving with the canonical HRF and then modulated with RT of each event for both the preceding car braking and crossing pedestrian conditions. In this analysis, positive and negative correlations indicated brain areas related to distraction and to the facilitation of the hazard detection response, respectively. Additional regressors were as follows: pedestrian crossing, preceding car slowdown, button press by the right thumb for hazard detection, button press by the left thumb to turn the wheel to the right, button press by the left thumb to turn the wheel to the left, scenario switch, and error (no-response event). These events were convolved with the canonical HRF. In addition, as in the TTLC analysis, we divided the run in half and examined the differences in activity between the first and second halves.

**Figure 2:**
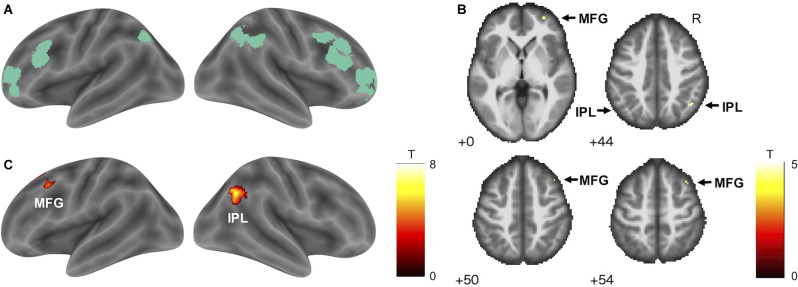
Frontoparietal control network and its positive correlation with TTLC. **(A)** The frontoparietal control network ROI (green) based on Gordon et al. (2016). **(B)** The result of hypothesis-based ROI analysis. Within the ROI, activity in the bilateral IPL and right MFG was positively correlated with TTLC, an index of driving accuracy (SVC; voxel-level threshold p_FWE_ < 0.05). Arrows indicate significant voxels. Numbers in each axial section indicate the *z* coordinates in MNI space. **(C)** Exploratory whole-brain analysis showed significant positive correlations between activity in the right IPL and left MFG with TTLC (cluster-defining threshold: *p* < 0.001 uncorrected; cluster-extent threshold: p_FWE_ < 0.05). Abbreviations: ROI, region of interest; TTLC, the time to line crossing; IPL, inferior parietal lobule; MFG, middle frontal gyrus; SVC, small-volume correction; FWE, family-wise error; MNI, Montreal Neurological Institute.

After the parameter estimation for each regressor in each participant, statistical inference for each regressor of interest was performed with a between-subjects (random effects) model using a one-sample *t*-test. Both positive and negative correlations were examined. Based on our *a priori* hypothesis that the frontoparietal control network would be involved in driving accuracy, a region of interest (ROI) analysis using a small-volume correction (SVC) implemented in SPM12 was applied to the analysis of positive correlations. A frontoparietal control network ROI was created based on a study that examined cortical parcellation from resting state functional connectivity (Gordon et al., 2016). From 24 coordinates comprising the frontoparietal control network, two lateral temporal regions, and two middle cingulate regions were removed to specifically focus on the core of the frontoparietal control network (Cole et al., 2013; Uddin et al., 2019). The coordinates of frontoparietal control network ROIs are shown in Supplementary Table 1. The 8-mm-radius spheres centered on the reported MNI coordinates of the frontoparietal control network were first created using **MarsBaR**^1^ and then combined. Finally, an intersection between the 20 spherical ROI masks and brain masks (mask.nii) derived from the current participants was created to exclude the area outside the functional image. The core frontoparietal control network ROI is shown in Figure 2A. In the other analyses, a whole-brain voxel-by-voxel analysis (one-sample *t*-test, cluster-defining threshold: *p* < 0.001 uncorrected; family-wise error (FWE) cluster-extent threshold: p_FWE_ < 0.05) was conducted to identify relevant areas across the entire brain.

## Results

### Participant Behavior

On average, 1.3% (SD = 1.4) and 0.6% (SD = 1.0) of trials responding to a preceding car slowing down and a crossing pedestrian, respectively, were miss trials. The mean RT of the hit trials for the preceding car and crossing pedestrian conditions was 0.81 s (SD = 0.03) and 0.95 s (SD = 0.04), respectively. The RT for the slowdown of a preceding car was significantly shorter than that for a crossing pedestrian (*t*_(29)_ = 4.94, *p* < 0.001). The mean coefficient of variation (CV) of RT for the preceding car and crossing pedestrian conditions was 0.32 (SD = 0.06) and 0.42 (SD = 0.08), respectively. The CV of RT for crossing pedestrian was significantly larger than that for the preceding car (*t*_(29)_ = 6.63, *p* < 0.001), indicating greater RT variability for crossing pedestrian condition. The time courses of TTLC and curvature averaged across the participants are shown in Supplementary Figure 1.

Correlation analysis between RT for pedestrian crossing and average TTLC 10 s before the event showed significant negative correlations in 16 out of 30 participants (*p* < 0.05), and two participants were still significant after Bonferroni’s multiple comparison correction. Correlation analysis between RT for preceding car braking and average TTLC in a period of 10 s before the event showed a significant negative correlation in 6 of 30 participants (*p* < 0.05), and no significant correlation was found when Bonferroni’s multiple comparison correction was performed.

### fMRI Results

#### (1) Driving Accuracy

A hypothesis-based ROI analysis targeting the frontoparietal control network revealed a significant positive correlation between TTLC (index of driving accuracy) and bilateral inferior parietal lobule and right middle frontal gyrus activity (Figure 2B, Table 1, SVC; voxel-level threshold p_FWE_ < 0.05). In the additional voxel-by-voxel whole-brain analysis, the right inferior parietal lobule and left middle frontal gyrus exhibited significant positive correlations (Figure 2C, Table 1, cluster-defining threshold: *p* < 0.001 uncorrected; cluster-extent threshold: p_FWE_ < 0.05).

**Table 1:** Brain regions correlated with TTLC.

Brain region		x	y	z	T	*p*_FWE_	cluster
*Positive correlation*				
Inferior parietal lobule*	R	46	−60	44	5.13	0.006	18
Inferior parietal lobule*	L	−40	−66	44	4.65	0.019	3
Middle frontal gyrus*	R	30	56	0	4.56	0.023	12
		38	22	54	4.36	0.037	3
		42	24	50	4.30	0.042	1
Inferior parietal lobule^†^	R	52	−64	36	8.48	0.008	494
		58	−56	32	5.87		
Middle frontal gyrus/	L	−38	22	52	6.01	0.025	370
superior frontal gyrus^†^							
		−20	36	52	5.73		
		−26	22	58	5.06		
*Negative correlation*				
Superior frontal gyrus	R	22	−4	64	16.27	0.000	66,954
		14	−54	62	15.97		
		22	−16	68	15.76		
Middle frontal gyrus	L	−28	40	22	6.50	0.001	762
		−28	36	32	6.31		

*Asterisks indicate the results of SVC analysis within the frontoparietal control network. Others indicate the results of whole-brain analysis. Daggers indicate a cluster that overlaps with the frontoparietal control network ROI. x, y, and z indicate the MNI coordinates. Abbreviations: TTLC, the time to line crossing; SVC, small-volume correction; FWE, family-wise error; MNI, Montreal Neurological Institute*.

**Figure 3:**
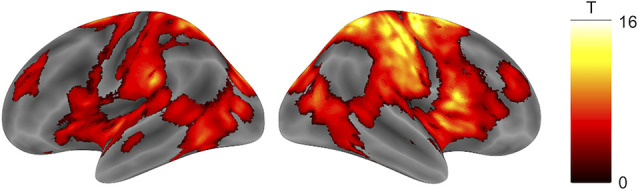
Brain activity negatively correlated with TTLC. Activity in wide-ranging bilateral cortical areas, especially the right sensorimotor cortex, was negatively correlated with TTLC (cluster-defining threshold: *p* < 0.001 uncorrected; cluster-extent threshold: p_FWE_ < 0.05). Abbreviation: TTLC, the time to line crossing.

There was a robust negative correlation between TTLC and broad cortical areas centered on the right sensorimotor cortex (Figure 3, Table 1, cluster-defining threshold: *p* < 0.001 uncorrected; cluster-extent threshold: p_FWE_ < 0.05).

A comparison of brain activity in the first and second half of the run revealed that the positive correlation between the frontoparietal control network and TTLC was stronger in the second half of the run (Table 2, SVC; voxel-level threshold p_FWE_ < 0.05). In the whole-brain analysis, the positive correlation between the inferior frontal gyrus that overlaps with the frontoparietal control network ROI and TTLC strengthened in the second half of the run as well (Table 2, cluster-defining threshold: *p* < 0.001 uncorrected; cluster-extent threshold: p_FWE_ < 0.05). The significant cluster also included the left hippocampus and left caudate nucleus. In the first half of the run, there was no region where the positive correlation with TTLC was stronger than the second half of the run.

**Table 2:** Brain activity differences between the first and second half of the task.

Brain region		x	y	z	T	*p*_FWE_	cluster
*TTLC positive correlation*				
First half > Second half				
no suprathreshold clusters				
Second half > First half				
Middle frontal gyrus*	L	−36	52	4	6.35	0.000	590
	L	−34	52	−2	6.30		
	L	−40	44	16	5.20		
	L	−20	58	−4	4.87		
	L	−24	50	4	4.77		
	L	−28	56	16	4.74		
	L	−36	42	8	4.68		
	L	−20	56	4	3.85		
Middle frontal gyrus*	R	30	58	0	4.89	0.016	174
	R	34	58	14	4.80		
	R	28	62	10	4.72		
	R	26	48	6	3.70		
Middle frontal gyrus*	L	−44	26	20	4.31	0.031	116
	L	−42	30	20	4.23		
							
Inferior frontal gyrus^†^	L	−26	22	−20	6.96	0.000	7,133
	L	−34	54	2	6.75		
	L	−42	16	−14	6.48		
Dorsomedial		0	26	44	4.89	0.046	342
prefrontal cortex					
	L	−2	50	44	4.14		
		0	40	54	4.10		
Cuneus	L	−4	−90	18	4.77	0.000	1,140
	R	2	−82	14	4.74		
	L	−6	−80	28	4.42		
Superior frontal gyrus	L	−18	38	46	4.64	0.025	415
	L	−22	44	40	4.62		
	L	−20	20	56	4.43		

*Asterisks indicate the results of SVC analysis within the frontoparietal control network. Others indicate the results of whole-brain analysis. Daggers indicate a cluster that overlaps with the frontoparietal control network ROI. x, y, and z indicate the MNI coordinates. Abbreviations: TTLC, the time to line crossing; SVC, small-volume correction; FWE, family-wise error; MNI, Montreal Neurological Institute*.

#### (2) Braking Response to a Preceding Car and a Crossing Pedestrian

**Figure 4:**
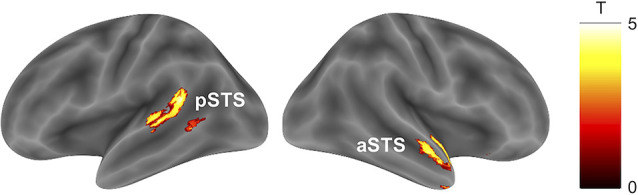
Brain activity associated with the braking response to a pedestrian crossing. Activity in the left pSTS and right aSTS preceding a pedestrian crossing was positively correlated with the RT for that event (cluster-defining threshold: *p* < 0.001 uncorrected; cluster-extent threshold: p_FWE_ < 0.05). Abbreviations: pSTS, posterior superior temporal sulcus; aSTS, anterior superior temporal sulcus; RT, reaction time.

The parametric modulation analysis with RT representing the braking response to a preceding car showed no significant clusters for positive and negative correlations representing distraction and facilitation processes, respectively (Table 3, cluster-defining threshold: *p* < 0.001 uncorrected; cluster-extent threshold: p_FWE_ < 0.05). The parametric modulation analysis using RT as a braking response to a crossing pedestrian revealed significant positive correlations in the right anterior superior temporal sulcus and left posterior superior temporal sulcus, indicating distraction (Figure 4, Table 3, cluster-defining threshold: *p* < 0.001 uncorrected; cluster-extent threshold: p_FWE_ < 0.05). The analysis of negative correlations revealed no significant clusters.

**Table 3:** Brain regions predicting the braking response to a preceding car slowing down or a crossing pedestrian.

Brain region		x	y	z	T	*p*_FWE_	cluster
Preceding car				
*Positive correlation*				
no suprathreshold clusters				
*Negative correlation*				
no suprathreshold clusters				
Pedestrian crossing				
*Positive correlation*				
Posterior superior	L	−60	−50	10	5.40	0.014	505
temporal sulcus	
		−64	−32	4	5.03		
		−56	−64	10	3.74		
Anterior superior	R	50	−10	−14	4.81	0.010	552
temporal sulcus	
		48	0	−20	4.41		
		30	16	−22	4.22		
*Negative correlation*					
no suprathreshold clusters					

*x, y, and z indicate the MNI coordinates. Abbreviations: FWE, family-wise error; MNI, Montreal Neurological Institute*.

There was no significant difference in brain activity predicting response to a preceding car and a crossing pedestrian between the first and second half of the run.

There was extensive activity in motor-related regions corresponding to button pressing with the right thumb in response to both preceding car braking and pedestrian appearing (Table 4, cluster-defining threshold: *p* < 0.001 uncorrected; cluster-extent threshold: *p*_FWE_ < 0.05). In addition, the preceding car braking recruited more activity in the cuneus and bilateral superior temporal gyrus compared to the pedestrian crossing (Preceding car braking > Pedestrian crossing contrast). On the other hand, in the contrast of Pedestrian appearing > Preceding car braking, there was activity around the inferior occipital gyrus and premotor cortex.

**Table 4:** Brain regions associated with preceding car braking and pedestrian appearing.

Brain region		x	y	z	T	*p*_FWE_	cluster
Preceding car braking					
Precentral gyrus	L	−42	−18	56	9.27	0.000	1,739
	L	−34	−24	48	6.97		
Premotor cortex	R	46	−2	44	6.37	0.000	1,126
	R	38	−14	46	6.11		
	R	32	−2	50	5.17		
Supplementary	R	8	12	54	6.33	0.000	2,826
motor cortex					
	R	16	−8	64	6.27		
	R	10	0	66	5.59		
Pedestrian appearing					
Precentral gyrus	L	−42	−16	54	8.29	0.000	6,491
	R	32	−2	50	7.56		
	R	8	14	56	7.32		
Preceding car braking >					
Pedestrian appearing					
Cuneus		0	−80	12	6.21	0.000	750
Superior temporal gyrus	R	54	−18	0	5.80	0.000	1,700
	R	42	−18	−10	5.35		
	R	62	−20	12	5.29		
Superior temporal gyrus	L	−60	−28	6	5.21	0.001	708
	L	−44	−18	−8	4.55		
	L	−44	−34	14	4.39		
Pedestrian appearing >					
Preceding car braking					
Inferior occipital gyrus	L	−46	−72	2	11.18	0.000	7,129
	R	48	−66	2	8.47		
	R	18	−72	50	7.49		
Premotor cortex	R	36	−2	50	7.74	0.000	1,560
	R	24	0	52	7.52		
	R	26	0	64	6.20		

*x, y, and z indicate the MNI coordinates. Abbreviations: FWE, family-wise error; MNI, Montreal Neurological Institute*.

## Discussion

Using a pedestrian-rich environment, we explored the neural activation associated with three types of safe driving performance. Driving accuracy was associated with higher activation of the bilateral frontoparietal control network and lower activation of bilateral extensive sensorimotor cortices. Activation of the left posterior and right anterior superior temporal sulci preceding the sudden crossing of a pedestrian was associated with a longer RT (slower braking response). We thus successfully identified neural correlates predicting lane-keeping and hazard detection in a pedestrian-rich environment.

The association of driving accuracy with activation of the frontoparietal control network identified here is supportive of the expected role of this network in driving safety. Previous studies have suggested a significant shift in activation from the occipital to the frontoparietal network under dual-task conditions (simulated driving plus secondary tasks) as compared to simulated driving only (Graydon et al., 2004; Just et al., 2008; Hsieh et al., 2009; Uchiyama et al., 2012; Schweizer et al., 2013; Chung et al., 2014). However, the relationship between the frontoparietal control network and safe driving performance has not been investigated previously, and the question of which aspects of driving are associated with the frontoparietal control network has remained unanswered. When this network was active, the car remained closer to the middle of the road and was less likely to run off the road. Conversely, with low activity in this network, the driver moved along the edge of the road, increasing the risk of going off the road. The frontoparietal control network is involved in executive functions (Niendam et al., 2012; Uddin et al., 2019), and activity in this network is considered to enable accurate vehicle control by maintaining attention despite distractions.

The clusters identified in the whole-brain analysis have overlapped with the frontoparietal control network ROI, but the activation peaks are located outside of the ROI. One way to think about this is that this region is also part of the frontoparietal control network involved in executive functions. This possibility is supported by previous studies that consider more dorsal MFG and ventral IPL as frontoparietal control network (e.g., Fischer et al., 2016). ROI selection may have also influenced this result. As shown in Supplementary Table 1, the current frontoparietal control network ROI based on a study conducted by Gordon et al. (2016) included rather right lateralized regions, which may have caused a small overlap between the ROI and whole-brain analysis. On the other hand, we might want to consider the possibility of this area having a different function such as the default mode and top-down attention since these regions have relationships with the default mode and dorsal attention networks depending on the subregion (Dixon et al., 2018).

In the analysis investigating the effect of learning/habituation, we identified the extensive lateral prefrontal activity that is associated with executive function as well as the left hippocampus and caudate nucleus in the second half of the run compared with the first half of the run. The hippocampus and caudate are known to involve episodic/spatial memory (Burgess et al., 2002) and motor learning (Jueptner et al., 1997), respectively. On the contrary, there was no area where the positive correlation with TTLC was stronger in the first half than in the second half. These results suggest that the relationship between brain activity and TTLC became clearer in the latter half of the task, which may have been caused by the fluctuation of both becoming larger due to fatigue or some kind of learning.

On the other hand, we are cautious in associating the identified relationships between the driving accuracy and decreased activation of sensorimotor cortices with driving safety. The activation of these areas has been associated with simulated driving itself (Walter et al., 2001; Uchiyama et al., 2003; Kan et al., 2013), and it is hard to imagine that sensorimotor processes would degrade driving accuracy. It would be more plausible to interpret the negative correlation with the sensorimotor cortices as reflecting a situation in which the driver is more engaged in recovering from the risk of approaching the edge of the road due to inaccurate driving. This interpretation is also supported by a prominent finding in the right sensorimotor cortices that could reflect control of the left thumb positioned on the steering buttons. Another possibility is that the decreased sensorimotor activity when TTLC is high reflects the reallocation of neural resources to executive functions (Bunge et al., 2000).

The pedestrian-rich environment enabled us to identify the association between activation of the bilateral temporal cortices and a delayed braking response to a crossing pedestrian for the first time. The finding seems in line with the conceptual framework holding that the processing load of a distractor reduces the detection of important environmental information and thereby driving safety (Marciano and Yeshurun, 2012, 2015; Murphy et al., 2016). The most distinctive feature of this study is the presence of social information in the form of a large number of pedestrians in the driving simulator environment. The lateral temporal lobe is associated with the processing of social information (Lahnakoski et al., 2012; Pitcher and Ungerleider, 2021); more specifically, the posterior part is associated with the perceptual component of social processing (Allison et al., 2000), whereas the anterior part is more closely related to higher semantic processing (Zahn et al., 2007; Binney et al., 2016; Oba et al., 2020). Because pedestrians are one of the major external distractors that capture a driver’s attention (Dingus et al., 2006), it is believed that such a stimulus could cause a task-irrelevant processing load for social information ranging from perceptual to higher semantic processing. The current behavioral findings such that the RT and CV of RT for pedestrian crossing are significantly longer and larger than that for the preceding car braking may support this consideration. This finding showed for the first time that the processing load associated with a pedestrian as a distractor is actually associated with a decrease in safe driving performance.

We identified no neural predictors of the braking response to a preceding car slowing down. One of the few studies in this area showed a positive correlation between car-following performance and activation of the bilateral lateral occipital complex and right inferior parietal lobule (Uchiyama et al., 2012). Similar findings were not obtained in the present study, probably due to differences in tasks and analyses. One reason for the lack of significant findings is that various influencing factors are involved in the braking response to a preceding vehicle, and the respective degrees of influence may be different for each individual. The difference between these results and those for the pedestrians may be because a pedestrian crossing the street causes the appearance of clear stimuli in the peripheral visual field, whereas a preceding car slowing down causes little change in terms of visual stimuli in the central field. Another possibility is that the intensity of the preceding car as a distractor is lower than that of the crossing pedestrian. However, the fact that the RTs of preceding car braking were faster than those of crossing pedestrians suggests that the awareness of brakes is greater, so the possibility that the difference in intensity affected the results may not be positively supported. It may also be possible that as shown in Supplementary Figure 2, RT for cars has a smaller fluctuation than RT for pedestrian crossing, and the parametric modulation used in this study may not have been able to detect a correlation with brain activity.

The negative correlation between RT and TTLC observed in some participants, especially in the pedestrian crossing condition, suggests that RT and TTLC may be under the control of the same attentional process. However, since there were differences in the relationship between RT and TTLC among conditions and individuals, we believe that the relationship between changes in attentional state and driving performance needs to be further investigated in the future. In addition, this result does not deny the existence of an interfering effect of social cognitive processing, which was discussed in the superior temporal sulcus, and it is possible that multiple last-minute factors may be involved in the response to a hazardous event.

The brain responses to the two dangerous events targeted in this study were different. The activity in the cuneus and bilateral superior temporal gyrus in the contrast of preceding car braking > pedestrian appearing may reflect the increased attention to the preceding car near the center of the visual field and the change in engine sound when the car decelerates in response to the preceding car braking. On the other hand, in the contrast of Pedestrian appearing > Preceding car braking, there was activity around the inferior occipital gyrus and premotor cortex. The Inferior occipital gyrus is a region corresponding to the extrastriate body area (Downing et al., 2001) and the body form area (Moro et al., 2008), suggesting that body form recognition and motor planning were more enhanced in pedestrian appearing.

This study has several limitations. Because the participants were young people, mainly university students who had been driving for just a few years, it is unclear whether the same results would be obtained for more experienced drivers or elder drivers. To confirm the general applicability of the results of this study, it is important to conduct further experiments with groups with different driving proficiencies and of different ages. From another point of view, the simulator is different from a real car, so it may be closer to the reaction (if they are different) of a driver who is not very familiar with driving, rather than a skilled driver. From the perspective of ecological validity, we believe that this study has various limitations. The car was controlled using a response button box, which is different from a car steering wheel. Therefore, the results of the current study might have been influenced by the response button. It would be important to confirm whether the frontoparietal control network and driving accuracy are positively correlated in an experiment using an MRI-compatible steering wheel as well. In a real driving situation, hazard situations rarely occur. Although we considered this point when we designed the study, we decided that it was impossible to prioritize ecological validity because it would require a very long experimental time. Therefore, in this study, we decided to maximize the number of trials within the limited experimental time. However, even within this limitation, we tried to prevent habituation as much as possible by randomly placing hazardous events such as pedestrians crossing from the right, crossing from the left, and braking of the vehicle ahead. Furthermore, by imposing the task of maintaining the center of the road, we tried to make it a dual task similar to actual driving. Regarding braking, the button-press response with the thumb is certainly different from the braking response with the leg. Therefore, it is possible that the cognitive load was different because the driving operation was not familiar to them. Actually, the fact that the driving operation and the interaction with the environment are not the same as in reality is a limitation of any simulator experiment. However, the real driving environment is also affected by individual differences in driving habituation, the state of the driver and the car, and the diversity of the driving environment. Given this variability of cognitive contexts in actual driving situations, we recognize that the present results are also generalizable findings as individual differences in driving characteristics in actual driving environments. The effect of unfamiliarity with the operation is likely to be mostly in the cognitive processes involved in the independently modeled driving operation and is unlikely to be substantially correlated with the accuracy of lane-keeping or the state of attention to the preceding car or pedestrians. The binary nature of button pressing does not allow us to examine the graded effect of braking. It is true that in actual driving, when there is a risk, we put our foot on the brake and increase the strength of the brake, considering both the expected time delay and the probability of the event. In this experiment, we were not able to analyze the attentional states involved in the dynamic process of braking, and our findings probably reflect the attentional states involved in the initial detection of risk. Finally, although there are other important driving performance measures for safe driving such as collisions, speed violations, and traffic light violations, in this study we targeted the RT and TTLC as representatives of driving performance (Greenlee et al., 2018; Akamatsu, 2019). In order to further clarify the relationship between safe driving and brain activity, it is necessary to examine the relationship with other driving performance measures.

Our overall results suggest the involvement of different cognitive processes in different components of driving safety: a facilitatory effect from maintained cognitive control on driving accuracy and a distracting effect from social–cognitive processes on the braking response to pedestrians. Research using a more realistic driving simulator that includes more factors is needed to elucidate the various cognitive processes that support and inhibit driving safety (Calhoun and Pearlson, 2012).

## Data Availability Statement

The raw data supporting the conclusions of this article will be made available by the authors, without undue reservation.

## Ethics Statement

The studies involving human participants were reviewed and approved by Research Ethics Committee of Tohoku University School of Medicine. The patients/participants provided their written informed consent to participate in this study.

## Author Contributions

KO, KH, AT-I, FM, MH, RK, and MS contributed to the conception and design of the study. KO and AT-I carried out the experiment. KO analyzed the data. KO, AT-I, and MS wrote the manuscript. All authors contributed to the article and approved the submitted version.

## Conflict of Interest

The authors declare that this study received funding from DENSO CORPORATION. The funder was not involved in the study design, collection, analysis, interpretation of data, the writing of this article or the decision to submit it for publication.

## Publisher’s Note

All claims expressed in this article are solely those of the authors and do not necessarily represent those of their affiliated organizations, or those of the publisher, the editors and the reviewers. Any product that may be evaluated in this article, or claim that may be made by its manufacturer, is not guaranteed or endorsed by the publisher.

## Footnotes

^1^ http://marsbar.sourceforge.net/
