# Supplementary material for: Neural Correlates Predicting Lane-Keeping and Hazard Detection: An fMRI Study Featuring a Pedestrian-Rich Simulator Environment
Source: Front Hum Neurosci. 2022 Feb 9;16:754379. doi: 10.3389/fnhum.2022.754379 (PMC8864087; doi:10.3389/fnhum.2022.754379)
Supplement: Supplementary file 1 [file Data_Sheet_1.DOCX]

Supplementary information

Supplementary Figure 1. The time courses of TTLC and curvature averaged across the participants.

The TTLC and curvature represented expected pattern from the S-shaped road that was repeated for three cycles in one session. Since the curvature was calculated based on the position of the vehicle, there was small variation in the large waveform of curvature. Abbreviation: TTLC, time to line crossing

Supplementary Figure 2. Example of HRF model for TTLC and hazard events

Abbreviations: HRF, hemodynamic response function; TTLC, time to line crossing; pm, parametric modulation

Supplementary Table 1. Coordinates of frontoparietal control network ROIs

|  |  |
| --- | --- |
|  | MNI coordinates (x y z) |
| L | -38.1 48.8 10.5 |
| L | -40.3 50.4 -4.8 |
| L | -34.1 -61 42.4 |
| L | -43 19.4 33.5 |
| L | -40.2 23.6 23.3 |
| L | -21.3 63.1 1.9 |
| L | -28.6 50.9 10.1 |
| R | 47.9 -42.5 41.5 |
| R | 38.1 45.9 7.7 |
| R | 42.8 48.3 -5.1 |
| R | 41.5 -53.5 44 |
| R | 35.7 -56.7 45.2 |
| R | 37.8 28.7 35.6 |
| R | 41.8 29.1 21.6 |
| R | 38.6 18.8 25.5 |
| R | 28.4 57 -5.1 |
| R | 23.5 59.1 4.9 |
| R | 30.9 52.2 9.9 |
| R | 42.4 19.5 48.2 |
| R | 38.9 9.6 42.7 |

These coordinates are based on a study conducted by Gordon et al. (Gordon et al., 2016).

Reference

Gordon, E. M., Laumann, T. O., Adeyemo, B., Huckins, J. F., Kelley, W. M., and Petersen, S. E. (2016). Generation and Evaluation of a Cortical Area Parcellation from Resting-State Correlations. *Cereb. Cortex* 26, 288–303. doi:10.1093/cercor/bhu239.
